# The experiences, challenges, and acceptance of e-learning as a tool for teaching during the COVID-19 pandemic among university medical staff

**DOI:** 10.1371/journal.pone.0248758

**Published:** 2021-03-26

**Authors:** Marwa Mohamed Zalat, Mona Sami Hamed, Sarah Abdelhalim Bolbol

**Affiliations:** 1 Department of Community, Environmental and Occupational Medicine, Faculty of Medicine, Zagazig University, Zagazig, Egypt; 2 Department of Family and Community Medicine, College of Medicine, Taibah University, Medina, KSA; National Taiwan University of Science and Technology, TAIWAN

## Abstract

**Background:**

e-learning was underutilized in the past especially in developing countries. However, the current crisis of the COVID-19 pandemic forced the entire world to rely on it for education.

**Objectives:**

To estimate the university medical staff perceptions, evaluate their experiences, recognize their barriers, challenges of e-learning during the COVID-19 pandemic, and investigate factors influencing the acceptance and use of e-learning as a tool teaching within higher education.

**Methods:**

Data was collected using an electronic questionnaire with a validated Technology Acceptance Model (TAM) for exploring factors that affect the acceptance and use of e-learning as a teaching tool among medical staff members, Zagazig University, Egypt.

**Results:**

The majority (88%) of the staff members agreed that the technological skills of giving the online courses increase the educational value of the experience of the college staff. The rate of participant agreement on perceived usefulness, perceived ease of use, and acceptance of e-learning was (77.1%, 76.5%, and 80.9% respectively). The highest barriers to e-learning were insufficient/ unstable internet connectivity (40%), inadequate computer labs (36%), lack of computers/ laptops (32%), and technical problems (32%). Younger age, teaching experience less than 10 years, and being a male are the most important indicators affecting e-learning acceptance.

**Conclusion:**

This study highlights the challenges and factors influencing the acceptance, and use of e-learning as a tool for teaching within higher education. Thus, it will help to develop a strategic plan for the successful implementation of e-learning and view technology as a positive step towards evolution and change.

## Introduction

COVID-19, a public health crisis of worldwide importance, was announced by the World Health Organization (WHO) in January 2020 as a new coronavirus disease outbreak and was reported as a pandemic in March 2020 [1].

Egypt reported the first German tourist death due to the virus on March 8. The increase in the number of cases to more than 100 cases by mid-March forced the government to make more rigid regulations. For one month, Egypt closed schools and universities and facilitated online distance electronic learning (e-learning) [2].

The pandemic of COVID-19 caused several schools and colleges to remain temporarily closed. Face-to-face education has ended by numerous schools, universities, and colleges. This will have negative impacts on educational activities, as social distance is crucial at this stage. Educational agencies are trying to find alternatives ways to manage this difficult circumstance [3]. This shutdown stimulated the growth of online educational activities so that there would be no interruption to education. Many faculties have been involved in how best to offer online course material, involve students, and perform evaluations [4].

This crisis would make the new technology accepted by organizations that were previously resistant to adapt. This was a difficult time for the educational sectors to deal with the current situation; professional education, particularly medical education, was more challenging [5].

Online e-learning is described as learning experiences using various electronic devices (e.g. computers, laptops, smartphones, etc.) with internet availability in synchronous or asynchronous environmental conditions. Online e-learning could be a platform that makes the process of education more student-centered, creative, and flexible [6]. Online delivery of courses is cost-effective and easily accessible especially when delivering curriculum to students in rural and remote areas [3]. The United online e-learning is seen by the United Nations (UN) and the WHO as a helpful tool for meeting educational needs, especially in developing nations [7]. Medical colleges have implemented numerous creative strategies to combat the crisis, using various software/apps such as Google Classroom, Zoom, and Microsoft Teams to take online courses. In order not only to complete the course but also to stay in constant contact with the learners, this virtual class of e-learning was initiated to grow the certainty and confidence of the students in their faculty during the COVID-19 pandemic [5].

It is anticipated that with the implementation of e-learning, the role of faculty members will be transformed from the traditional teacher-centric to student-centric model which serves the current new curriculum applied at our college of medicine. Therefore, this study aims to estimate the university staff perceptions, evaluate their experiences, recognize their barriers, and assess their challenges to e-learning during the COVID-19 pandemic. Additionally, the study will investigate factors influencing the acceptance of e-learning as a tool for teaching within higher education which could help future endeavors aimed at implementing e-learning not only during the pandemic but in other non-pandemic situations throughout the teaching life.

## Materials and methods

### Study design and setting

A cross-sectional study was conducted from September 1st to October 1st, 2020 at the Faculty of Medicine, Zagazig University, Sharkia Governorate, Egypt.

### Study population and sample size

The medical staff of both basic science and clinical departments who are engaged in the development and teaching of online courses were invited to participate in the study. While, those who refused participation, retired, or on leaves (e.g. sick, maternity, or any type of leaves) were excluded.

The required sample size was calculated to be 346 staff members. Calculations have been done using the sample size software online for prevalence studies [8]: the total number of staff members in both basic science departments (i.e. anatomy, physiology, pathology, histology, biochemistry, parasitology, pharmacology, microbiology), and clinical departments (i.e. internal medicine, surgery, gynecology & obstetric, pediatrics, community medicine, family medicine …..etc.) was 3439 at the faculty of Medicine, Zagazig university at the time of the study, assuming a prevalence of 50%, a precision of 5% at confidence interval 95% and power of test 80%.

### Tools of data collection

A semi-tailored electronic questionnaire was used and contains four parts:

**First Part**: questions on ***socio-demographic and occupational data*** of the participants as age, gender, marital status, residence, work sector (academic or clinical), current employment status, years of teaching experience, whether they have taught an online course before or not, and their experience duration.

**Second part**: questions on ***university staff perceptions and experiences of online courses*** adapted from a previous study [9]. The questions are rated on a 5-point scale ranging from strongly disagree = 1 to strongly agree = 5 by which the staff member could express their agreement levels.

**Third Part**: questions on ***barriers and challenges*** towards online learning. Medical staff should rank the challenges facing distance education in order of their seriousness (1–10 scale, 1 being the least serious, 10 being the most serious) [10].

**Fourth part:** questions based on the validated Technology Acceptance Model (TAM) [11], for exploring factors that affect university medical staff ***acceptance and use of e-learning*** as a teaching tool. It consisted of three items namely perceived usefulness, perceived ease of use, and acceptance on a 5-point scale ranging from ‘‘strongly disagree” to ‘‘strongly agree.”, Acceptance was categorized as accept and don’t accept according to the median (median = 2.5), scores above 2.5 indicate acceptance while rated scores <2.5 indicate refusal.

Data analysis techniques used for detection of the percentage of respondents’ response is described in detail in the work of Napitupulu et al. [12] and the range of results compared to the following categories: 0–25% Strongly Disagree, 26–50% Disagree, 51–75% Agree, 76–100% Strongly Agree.

### Procedures of data collection

The electronic questionnaire was designed on Google forms, and the invitation link for participation in the survey was shared via mail and on social media such as each department WhatsApp group, by the researchers, through the departments’ coordinators. Another two reminders were sent every 10 days to increase the participants’ response rate. A cover letter was presented on the first page of each electronic survey explaining the purpose of the study, emphasizing its importance and significance, therefore encouraging cooperation by the respondents.

### Pilot study

The questionnaire was tested on 10 staff members. The necessary modifications, changes, and corrections were done to ensure ease of understanding and clarification of all questions. For testing the questionnaire reliability, Cronbach’s alpha test was used and was >0.70 for most of the items.

### Data management

Data were analyzed using the SPSS version 20.0. The Shapiro-Wilk test was used to assess the normality of data distribution. Descriptive analysis was performed for quantitative data by mean, standard deviations and for qualitative data by frequencies and percentages as applicable. A Multivariate regression analysis was performed to predict potentially significant determinants of acceptance and use of e-learning in education. A P-value of < 0.05 was considered statistically significant.

### Ethical considerations

The necessary official permissions were obtained from the Zagazig University Institutional Review Board (Ref No #6385-1-9-2020#). Consent from the participant after being informed about the purpose of the study and research objectives was obtained at the start of the online survey. Privacy and confidentiality were assured.

## Results

A total participant in this study was 346 university medical staff members. Most of the participants are females (87.9%) with a mean age of 47 years most of them are married (72%). Most of the staff members live in the same city where they work (76%) with a mean of 19 years of teaching experience, and more than half of them (63.9%) were from the basic science departments. Half of the teaching staff are professors (52%) and taught online courses before (40.2%) for more than 2 years and taught both theoretical and practical sessions (Table 1).

**Table 1:** Socio-demographic data of the studied group.

	University Medical Staff (346) No. (%)
Age (Mean±SD)	47.44±8.804
**Gender:**	
Male	42 (12.1)
Female	304 (87.9)
**Marital status:**	
Single	83(24.0)
Married	249 (72.0)
Widowed	14 (4.0)
**Residence:**	
Away from workplace	83 (24.0)
In the same city of workplace	263 (76.0)
Teaching Experiences (years) (Mean±SD)	19.86±9.543
**Work sector:**	
Basic science departments	221 (63.9)
Clinical departments	125 (36.1)
**Rank:**	
lecturer	14 (4.0)
Assistant professor	69 (19.9)
Associate professor	83 (24.0)
Professor	180 (52.0)
**Have you high internet speed at home:**	
No	28 (8.1)
Yes	318 (91.9)
**Have you ever taught a course online before Covid-19?**	
No	207 (59.8)
Yes	139 (40.2)
**If yes, in which areas?**	
Practical sessions*	23 (3.8)
Theoretical sessions**	56 (16.2)
Both	70 (20.2)
**If yes, what is the duration?**	
< 1year	42 (12.1)
1–2 years	28 (8.1)
>2 years	83 (24)

*Practical sessions (e.g. tutorial, problem-solving, case scenario discussion, photos, slides, x-rays imaging).

**Theoretical sessions (e.g. recorded lectures, videos).

Study results revealed that all the staff members agreed that the online course design permits staff to educate at their own speed (36.1% strongly agreed and 63.9% agreed), followed by 88% of the staff members agreed that the technological skills acquired from teaching online courses increased their educational experience (56.1% strongly agreed and 32.1% agreed). While 44.2% of staff members disagreed that tests in an online course are more difficult for students (4% strongly disagreed and 40.2% disagreed) compared to 43.9% agreement (7.8% agree and 36.1% strongly agree) (Table 2).

**Table 2:** Perceptions and experiences of university medical staff towards e-learning.

	University Medical Staff (346) No. (%)				
	Strongly agree	Agree	Neutral	Disagree	Strongly disagree
**One of the benefits of teaching an online course is flexibility.**	98 (28.3)	206 (59.5)	28 (8.1)		14 (4.0)
**In the classroom environment, face-to-face contact with students is favored over an online classroom setting.**	166 (48.0)	124 (35.8)	42 (12.1)	14 (4.0)	
**Practical courses in an online course are among the most difficult for medical staff members**	69 (19.9)	179 (51.7)	70 (20.2)	28 (8.1)	
**The online course design permits staff to educate at their own speed**	125 (36.1)	221 (63.9)			
**Theoretical courses should be offered online**	180 (52.0)	97 (28.0)	27 (7.8)	42 (12.1)	
**Online courses attract learners because there is no needed set up for the classroom**	111 (32.1)	165 (47.7)	42 (12.1)	28 (8.1)	
**The lack of student-to-student contact in an online class will minimize their experience of learning.**	56 (16.2)	192 (55.5)	14 (4.0)	84 (24.3)	
**Exams in an online course are harder for students**	27 (7.8)	125 (36.1)	41 (11.8)	139 (40.2)	14 (4.0)
**It’s harder to administer exams in an online course**	55 (15.9)	83 (24.0)	83 (24.0)	111 (32.1)	
**Online courses enable content self-learning more than “classic” face-to-face course**	27 (7.8)	221 (63.9)	56 (16.2)	42 (12.1)	
**The technical skills of an online course improve the educational efficiency of the college staff’s experience.**	194 (56.1)	111 (32.1)	27 (7.8)	14 (4.0)	
**Online courses require more discipline from students more than in conventional courses.**	95 (27.5)	168 (48.6)	42 (12.1)	41 (11.8)	

Applying the Technology Acceptance Model (TAM) to university medical staff members showed that the percentage of the respondent’s answer on perceived usefulness was 77.1%, this means that university medical staff found that e-learning is very helpful in improving and progressing the educational process. The percentage of the respondent’s answer on perceived ease of use was 76.5%, this means that users assess e-learning systems implemented by being highly easy to use and operate. While the percentage of the respondent’s answer on acceptance of e-learning was 80.9%, this means that based on user perception, the e-learning system implemented was with high acceptance level. This was obtained because perceived ease of use and perceived usefulness have been assessed to be adequate for the users (Table 3).

**Table 3:** Technology Acceptance Model results of university medical staff to e-learning.

Item	Questions	Percentage of response	Category
**Perceived usefulness**	Accelerate work	77.1%	Strongly Agree
	Improve performance		
	Increase productivity		
	Effective		
	Simplify work		
	Helpful		
**Perceived Ease of Use**	Easy to learn	76.5%	Strongly Agree
	Can be controlled		
	Clear and understandable		
	Flexible		
	Easy to use		
	Easy to be skilled		
**Acceptance of e-learning**	I will use e-learning in teaching in the future	80.9%	Strongly Agree
	I will use e-learning frequently		
	I am satisfied with e-learning		
	I recommend others to use e-learning		

Studying the barriers of e-learning as reported by the university staff members showed that (40%) reported insufficient/ unstable internet connectivity followed by inadequate computer labs (36%), lack of computers/ laptops (32%), and technical problems (32%) (Table 4).

**Table 4:** Barriers and challenges of e-learning to university medical staff.

Barriers and Challenges	Frequency (%)
Insufficient/ unstable Internet connectivity	139 (40.2)
Inadequate computer labs	125 (36.1)
Lack of computers/ laptops	111 (32.1)
Technical problems	111 (32.1)
Heavy workload of the online courses	98 (28.3)
Limited technology skills of the staff	84 (24.3)
Staff resistance and negative attitude towards e-learning	84 (24.3)
Level of interactions with students in the online course is lower than in a traditional face-to-face class.	84 (24.3)
Difficult applying distance learning for practical sessions and courses	83 (24.0)
Longer time to prepare for an online course	70 (20.2)
Lack of protection for the developed e-materials	69 (19.9)
Shortage of teaching staff	69 (19.9)
Lack of incentives/ Non-repayment for Internet outside the college	56 (16.2)
Difficulty for motivating the students in the online environment than in the traditional setting	56 (16.2)
Lack of suitable online environment at home (e.g. presence of children, other family members)	42 (12.1)
Difficult dividing students into subgroups for group task working	42 (12.1)
Difficult receiving student feedback in the online course versus in a traditional face-to-face class.	42 (12.1)

Statistical analysis was conducted to identify risk factors in terms of unadjusted OR. Teaching experience duration (years) followed by the online courses they taught before COVID-19, age of staff members (years), and work sector whether academic or clinical were the significant factors that influence acceptance of e-learning. A logistic regression analysis was done to study the significant independent factors affecting e-learning acceptance and showed that age under 40 years, teaching experience less than 10 years, and being a male are the most important indicators affecting e-learning acceptance (Table 5).

**Table 5:** Socio-demographic and occupational factors affecting acceptance of e-learning and results of logistic regression analysis.

Predictors	Unadjusted OR (95% CI)	Adjusted OR (95% CI)
**Age (<40 years)**	0.229 (0.053–0.986)*	9.889 (9.717–1.007)*
**Gender (Male)**	0.250 (0.033–1.891)	0.011 (0.000–0.254)*
**Duration of teaching experiences (<10 years)**	5.543 (2.468–12.450)*	41.248 (8.477–200.714)*
**Work sector (Academic)**	2.788 (1.032–7.528)*	1.029 (0.241–4.388(
**Having high internet speed**	1.406 (0.397–4.984)	1.491(0.259–8.562)
**Previous teaching an online course before Covid-19**	0.341 (0.152–0.764)*	0.935 (0.244–3.575)

* P < 0.05 is significance.

## Discussion

e-learning is not considered a new phenomenon, there was an increasing global trend of using electronic learning or e-learning in the last decade and some higher education institutes in developing countries have adopted this trend recently [13]. However, this technology has not been evenly dispersed throughout all nations and cultures [14].

More than nine months have passed since the WHO declaration of COVID-19 as a pandemic, with an abrupt shift to online teaching and electronic learning. Furthermore, the uncertain future concerning returning to normal life and stopping this pandemic results in maximum dependency on e-learning especially in higher education [15].

Like other countries, Egypt faced significant challenges in higher education and transferred its in-person educational system to virtual learning. A particular urgent challenge was for face-to-face university courses to be delivered online [16]. In this study, the e-learning perception, challenges, and predictors of its acceptance as a method for education during the COVID-19 pandemic were investigated among the university medical staff members.

The majority of the participants agreed (32.1%) and strongly agreed (56.1%) that the technological skills to provide online courses increase the educational value of the experience of the faculty staff members. Similarly, these findings from our research support the results of previous studies [17–19].

The majority of our participants agreed (59.5%) on the advantages of time flexibility of teaching the online course. In contrast, other previous studies [19], reported that faculty members considered that e-learning can take time and can lead to student monitoring difficulties and can reduce the interest in direct traditional teaching.

These various perceptions might be related to unfamiliarity with the e-learning medium, different technological knowledge, and skills of the participants which highlight the need for formal training and workshops on using various technological methods and platforms for strengthening the e-learning activities.

The current study showed that 36.1% and 63.9% of the participants strongly agreed, and agreed respectively that the online course enables staff to teach at their own pace. Similarly, a previous study appreciated the self-pacing of online learning [20].

Also, most of our participants disagreed/ strongly disagreed (44.2%) that exams in an online course are harder for students. The reason for this staff perception might be attributed to the fact that most of the online tests are based on multiple-choice questions which allow testing a large number of students quickly, and across a vast expanse of content than essay questions. Furthermore, the automated marking of the tests saves the staff members efforts and time [21]. On the contrary, another study by Hannafin et al. [22] noted that many observational and participatory evaluations of distant learning were difficult. Likewise, Oncu & Cakir [23] noticed that because of the lack of face-to-face interaction, informal assessment can be challenging for online instructors. Nevertheless, there are indeed best practices and techniques for conducting assessments securely with a sort of protection system in the online environment.

In the present study, the application of the TAM on our participants revealed that a higher percentage of the respondents agreed with the perceived usefulness of e-learning which means that university medical staff accepts that e-learning is valuable in improving and progressing the teaching and learning process. Meanwhile, prior research by Poon et al. [24] reported that their participants at several local universities were not fully comfortable with e-learning as a tool for teaching and attributed this perception to many factors as technological challenges, difficult interactions and discussions with students, lack of adequate internet connectivity and personal learning preference [25].

Inconsistent with Choreki [26], our survey findings bring to light that most of the respondents agreed on the ease of use of e-learning which means that medical staff assesses e-learning systems implemented by being profoundly simple to use and operate. This could be attributed to the fact that our college was recently started their new blended learning program (i.e. the combination of e-learning technology with the traditional face-to-face teaching) short times before the COVID-19 pandemic with intensive training for all staff members on the online courses, planning and designing the teaching materials before its formal application for students.

In our college, both synchronous (live or in real-time) and asynchronous (recorded or self-paced) e-learning strategies were implemented through learning management systems (LMS) with their applications (e.g. Zoom and Microsoft Teams). Synchronous e-learning was offered in the form of interactive teaching and clinical case discussions in small and large group formats. Asynchronous e-learning included preparation of course materials for students in advance of students’ access (e.g. recorded lectures, supportive videos, external links for recommended websites, and additional resources such as electronic books). These enhance the staff adoption of the new technology and its integration into their teaching activities [19].

This study showed that the e-learning system was implemented with a high acceptance level. Several studies were done in different countries [27–29] reported that the user adoption and acceptance of e-learning were influenced by a diverse individual (e.g. readiness to use e-learning), social (e.g. interpersonal and instructor influence), and organizational (e.g. technological facilities, financial and infrastructure) factors within a specific culture, in addition to the perceived benefit and ease of use of e-learning systems.

Studying the barriers of e-learning as reported by our survey revealed that reported insufficient/ unstable internet connectivity, inadequate computer labs, lack of computers/ laptops, and technical problems were the highest challenge for adapting to e-learning. In alignment with these findings, recent research by Nguyen et al. [30] demonstrated that the main obstacles to e-learning are based on several stakeholder perspectives of infrastructure, technology, management, support, execution, and pedagogical aspects. Likewise, another study illustrated that e-learning tools should meet the users’ requirements to gain their trust and improve their acceptance of e-learning [31]. Additional study classified e-learning barriers into learners, teachers, curriculum, organizational and structural factors that need more collaboration for their solutions [32].

As regards the factors predicting the acceptance of e-learning, the logistic regression analysis showed that age under 40 years, teaching experience less than 10 years, and male gender are the most important indicators affecting e-learning acceptance. This could be clarified by the reality that younger staff already using technology in general than older, which would increase their abilities, willingness, and acceptance to use other e-learning technology. Furthermore, this result is in agreement with Fischer et al. [33] who stated that older staff with long traditional teaching experience usually has limited interaction with technology and lacking the development of their necessary skills.

Adamus et al. [34], reported women’s preference for accepting e-learning than men’s. In contrast, past studies showed unfavorable differences for women due to mental overload, stress, and difficulties with work-life balance [35, 36].

Meanwhile, other studies reported scarce differences between males and females in their use of e-learning, their motivation, and satisfaction [37]. The reason for this difference may be related to different gender representation in the studies.

### Limitation of the study

This study has some potential limitations. Being a cross-sectional study, the participants’ perceptions may change over time. Therefore, a further longitudinal study is required to enhance the understanding of determinants that are critical to the adoption of e-learning systems in our community. Also, the present study was conducted in one medical college. So, in the future, additional studies need to be done using subjects from other universities to assess the adoption and acceptance of e-learning in higher educational institutes.

## Conclusions

e-learning was underutilized in the past, especially in developing countries. However, the current crisis of the COVID-19 pandemic enforced the entire world to rely on it for education.

In the current study, the majority of participants strongly agreed with the perceived usefulness, perceived ease of use, and acceptance of e-learning. The highest challenge for accepting e-learning were insufficient/ unstable internet connectivity, inadequate computer labs, lack of computers/ laptops, and technical problems. The significant indicators affecting e-learning acceptance were age under 40 years, teaching experience less than 10 years, and male gender. This study highlights the challenges and factors affecting the acceptance of e-learning as a tool for teaching within higher education, in developing countries and may lead to strategic development and implementation of e-learning and view technology as a positive step towards evolution and change.

## Supporting information

S1 Dataset (SAV)
